# Adults with congenital heart disease: ready for mobile health?

**DOI:** 10.1007/s12471-019-1237-2

**Published:** 2019-02-13

**Authors:** R. W. Treskes, M. Koole, D. Kauw, M. M. Winter, M. Monteiro, D. Dohmen, A. Abu-Hanna, M. P. Schijven, B. J. Mulder, B. J. Bouma, M. J. Schuuring

**Affiliations:** 1 Department of Cardiology, Leiden University Medical Centre, Leiden, The Netherlands; 2 Department of Cardiology, Academic Medical Centre, Amsterdam, The Netherlands; 3 Department of Cardiology, Rode Kruis Ziekenhuis, Beverwijk, The Netherlands; 4 FocusCura, Driebergen-Rijsenburg, The Netherlands; 5 Department of Medical Informatics, Academic Medical Centre, Amsterdam, The Netherlands; 6 Department of Surgery, Academic Medical Centre, Amsterdam, The Netherlands; 7 Department of Cardiology, Haga Teaching Hospital, The Hague, The Netherlands

**Keywords:** Congenital heart disease, GUCH, Resource utilisation, Delivery of healthcare, Mobile health, eHealth

## Abstract

**Purpose:**

Mobile health (mHealth) could improve the outcome of grown-up patients with congenital heart disease (GUCH) and reduce their emergency care utilisation. Inappropriate use of mHealth, however, can lead to data overload for professionals and unnecessary data collection for patients, increasing the burden for both. We aimed to determine the clinical characteristics of patients with high emergency care utilisation and to test whether these patients were willing to start using mHealth.

**Methods:**

Clinical characteristics and emergency care utilisation of consecutive GUCH patients who visited one of the two participating cardiologists at the outpatient clinic of the Academic Medical Centre in Amsterdam were studied retrospectively. All patients were approached to fill in an mHealth questionnaire. A frequency of three or more emergency visits in 5 years was defined as high emergency care utilisation.

**Results:**

In total, 202 consecutive GUCH patients who visited one of the two participating cardiologists were studied. Median age was 41 years, 47% were male, and 51% were symptomatic. In the previous 5 years, 134 emergency visits were identified. Of all patients, 8% had high emergency care utilisation. High emergency care utilisation was associated with patients being symptomatic, using antiarrhythmic drugs or diuretics. In total, 75% of all patients with high emergency care utilisation were willing to start using mHealth.

**Conclusion:**

GUCH patients who are symptomatic, those on antiarrhythmic drug therapy and those on diuretics are suitable candidates for enrolment in future mHealth initiatives because of both high care utilisation and high motivation to start using mHealth.

## What’s new

- Telemonitoring with mobile phones is promising, but research remains to be done.
- Grown-up patients with congenital heart disease have a proven interest in mobile health.
- This study identifies the characteristics of patients with high healthcare use.
- The vast majority of these patients is in possession of a smartphone and willing to start using mobile health.

## Introduction

Congenital heart disease (CHD) is one of the most common birth defects [1–3]. During the past decades, the life expectancy of children born with a CHD has increased dramatically. At present, 95% of children with CHD reach adulthood [1]. However, many of the grown-ups with congenital heart disease (GUCH) are chronically affected by residual sequelae leading to unpredictable arrhythmias, heart failure and a reduced quality of life [4–8]. In general, GUCH patients have a high utilisation of emergency resources, with emergency care utilisation increasing as age progresses [4]. As the population of GUCH patients is increasing in number and age, total emergency care utilisation of this population is expected to increase [9].

Mobile health (mHealth) is the provision of medical care by mobile technologies capable of delivering health information, monitoring clinical signs and enabling direct care and patient education [10]. Using mobile technology, vital signs can be collected and sent immediately to a treating cardiologist. E‑visits enable immediate and remote contact between doctor and patient [11]. Therefore, potential benefits of mHealth include: rapid delivery of round-the-clock care; enhanced daily monitoring and hence timely response and more convenience for patients; and improved access for patients [12]. In order to improve outcome and reduce emergency care utilisation, careful selection of patients that are most likely to benefit from an mHealth intervention is warranted. If used in an inappropriate patient population, mHealth can lead to data overload for healthcare professionals and unnecessary data collection for patients, increasing the burden for both [13]. Patients with a high emergency care utilisation and high motivation to start using mHealth are suitable candidates to include in new mHealth initiatives. It is therefore the primary objective of this study to determine the clinical characteristics of GUCH patients with high emergency care utilisation. It is the secondary objective to combine these findings with the results of an mHealth questionnaire, to test whether GUCH patients with high emergency care utilisation are willing to start using mHealth.

## Methods

### Population and data collection

For this study, two cardiologists specialised in GUCH (B.B. and B.M.) approached consecutive patients who had an appointment at the outpatient clinic with them to fill in an mHealth questionnaire. These patients visited the outpatient clinic at the Academic Medical Centre in Amsterdam between April 2016 and September 2016. Clinical characteristics and emergency care utilisation of these GUCH patients were studied retrospectively. Clinical characteristics noted were: severity of the CHD (in accordance with the Bethesda conference) [14], history of cardiac surgery, history of pacemaker or implantable cardioverter defibrillator (ICD) implantation and the use of diuretics or any antiarrhythmic drug therapy. In patients receiving antiarrhythmic drug therapy, the indication was noted as well. Beta-blockers were considered an antiarrhythmic drug therapy if the drug was initiated or the dose was altered for symptoms of palpitations or treatment for arrhythmia control. Cardiac-related symptoms were rated in accordance with the New York Heart Association (NYHA) Functional Classification. GUCH patients with a NYHA class II or higher were considered symptomatic. Emergency care utilisation was defined as visits to the emergency room, cardiac care unit or unplanned outpatient clinic visits. Outpatient clinic visits were counted if they included a visit to a cardiologist, cardiologist in training, heart failure nurse or dedicated CHD nurse at the Department of Cardiology of the Academic Medical Centre. An outpatient clinic visit was considered ‘unplanned’ if the electronic medical record explicitly stated that the patient was seen without a scheduled appointment in case of symptoms. Interventions noted following an emergency care visit were any type of open-heart surgery, aneurysm surgery, pacemaker or ICD implantation or replacement, diagnostic catheterisations, electrical cardioversions (ECV), catheter-based interventions and bronchoscopy in case of haemoptysis. High care utilisation was defined as a score of three or more emergency visits between 1 June 2011 and 31 December 2016.

All patients were approached to fill in an mHealth questionnaire on paper. Details of the questionnaire have been described previously [15] Exclusion criteria were being mentally impaired (at physician’s discretion), having no knowledge of the Dutch language or being younger than 18 years of age.

### Data management and statistics

SPSS 22 (IBM Corp. Released 2013. IBM SPSS Statistics for Windows, Version 22.0. IBM Corp., Armonk, NY, USA) was used for statistical analysis. To identify GUCH patients who would most likely benefit from mHealth, determinants were set off against an emergency care utilisation of three or more emergency visits and/or interventions in the previous 5 years. Variables were compared with a chi-squared test. A *p*-value ≤ 0.05 was considered statistically significant.

## Results

### Population characteristics

In total, 202 consecutive patients who visited the outpatient clinic and had an appointment with one of the two participating cardiologists (B.B. and B.M.) at the Academic Medical Centre in Amsterdam between April 2016 and September 2016 were studied. Median age was 41 years (interquartile range 32–50, range 18–78 years), 47% were male and 51% were symptomatic. Of all patients, 19% had mild CHD, 61% moderate CHD and 20% severe CHD. A total of 83% had a history of cardiac surgery and 8% had had a pacemaker or ICD implanted. Thirty-one per cent received antiarrhythmic drug therapy and 9% used diuretics (Tab. 1). Only 5% were in NYHA class IV. All patients filled in the mHealth questionnaire.

**Table 1:** Comparison of high and low care utilisation. *PM *Pacemaker, *ICD* implantable cardioverter-defibrillator

	All patients (*n* = 202)	Low care utilisation (*n* = 186 (92%))	High care utilisation (*n* = 16 (8%))	*p*
Median age, years	41 (18–78)	40 (18–78)	42 (23–77)	
Male, %	94 (47)	87 (46)	7 (43)	0.816
*Congenital heart disease*				
Mild, %	39 (19)	35 (19)	4 (25)	0.548
Moderate, %	123 (61)	116 (62)	8 (50)	0.352
Severe, %	40 (20)	35 (19)	4 (25)	0.548
*New York Heart Association class*				
Class I, *%*	97 (48)	95 (51)	2 (13)	<0.001
Class ≥ II, *%*	105 (52)	91 (49)	14 (87)	<0.001
*Event history*				
Cardiac surgery, %	168 (83)	156 (83)	12 (75)	0.363
PM/ICD implantation, %	17 (8)	14 (8)	3 (19)	0.121
*Medication*				
Diuretics, %	19 (9)	12 (6)	7 (44)	<0.001
Antiarrhythmic, %	62 (31)	51 (27)	11 (69)	0.001
*mHealth*				
Smartphone utilisation (%)	186 (92)	172 (92)	14 (87)	0.369
Wiling to use mHealth (%)	143 (71)	131 (70)	12 (75)	0.70

### Emergency visits

In the previous 5 years, 202 patients accounted for 134 emergency visits, 59 (29%) of whom had one or more emergency visit. Sixteen (8%) of the 202 patients had high care utilisation and 186 (92%) low care utilisation. No significant differences in gender, history of cardiac surgery or severity of CHD were found between patients with high and low care utilisation. Significant differences were found in NYHA class (87% vs 49%, *p* < 0.001), use of diuretics (44% vs 7%, *p* < 0.001) and antiarrhythmic drug therapy (69% vs 27%, *p* = 0.001) (Tab. 1).

**Fig. 1:**
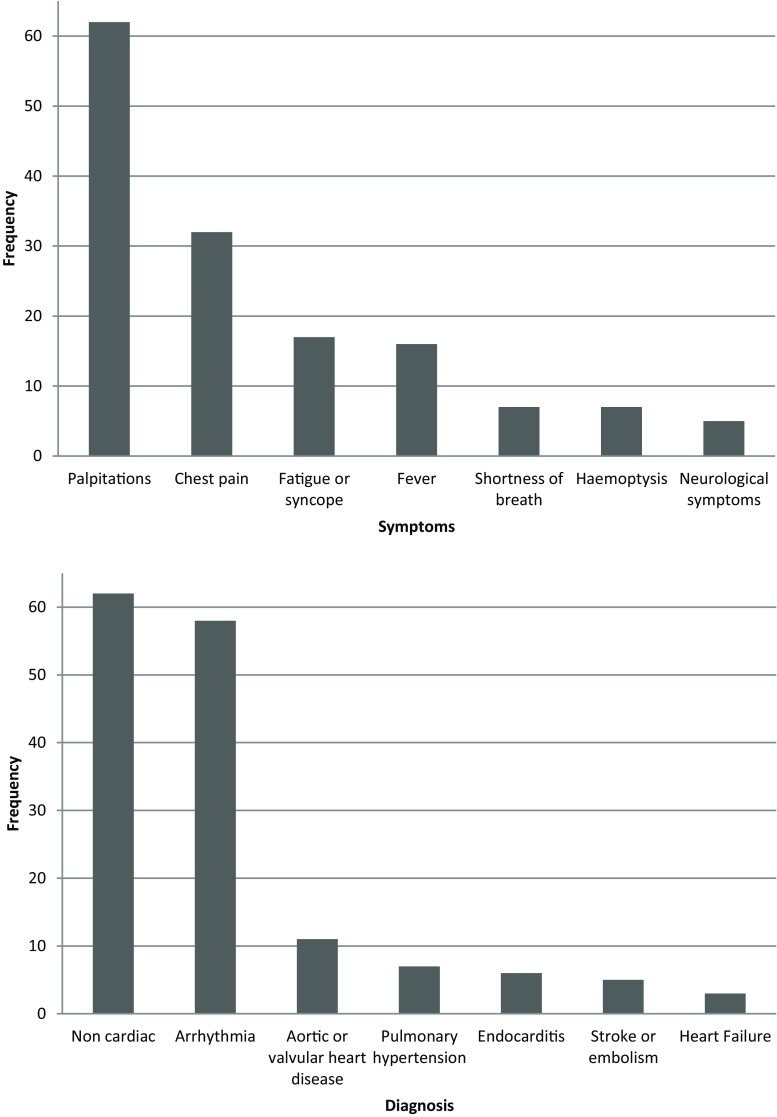
Frequency of emergency care visits, reasons and subsequent diagnoses

Tab. 2 and Fig. 1 show all of the symptoms with which patients presented, the subsequent diagnoses made, and the treatment administered. Most patients presented with either palpitations (41%) or chest pain (24%). In 46% of all cases, no diagnosis of a cardiac nature was made. In 37%, a patient was diagnosed with an arrhythmia (Fig. 1).

**Table 2:** Information on emergency visits

**Symptoms**	***n*** **(%)**
Palpitations	55 (41%)
Chest pain	32 (24%)
Fever	16 (12%)
Fatigue	13 (10%)
Shortness of breath	7 (5%)
Haemoptysis	6 (4%)
Neurological symptoms	5 (4%)
**Diagnoses**	
No diagnosis of cardiac nature	62 (46%)
Arrhythmia	50 (37%)
Endocarditis	6 (5%)
Pulmonary hypertension	6 (5%)
Stroke	5 (4%)
Valvular heart disease	3 (2%)
Heart failure	2 (1%)
**Therapeutic regimen consequences**	
No changes in therapeutic regimen	59 (44%)
Medication changes	52 (39%)
Electrocardioversion	29 (21%)
Interventions	4 (3%)
Planned interventions	3 (2%)

Emergency visits resulted in a variety of different actions. In 44% of all cases the therapeutic regimen was not changed. Drug therapy was changed in 52 (39%) cases. In 8 (15%) out of 55 cases of palpitations, the therapeutic regimen was not changed. Therapeutic regimen changes included 16 (29%) cases of ECV, 13 (24%) cases of adjusting antiarrhythmic drug therapy after ECV, 7 (13%) cases of adjusting antiarrhythmic drug therapy only, 10 (18%) cases of initiating antiarrhythmic drug therapy and 1 (2%) case of radiofrequency ablation. In 29 (91%) of the 32 cases of chest pain no action was taken. Therapeutic regimen changes included 3 (9%) cases of initiating antibiotic treatment for the suspicion of endocarditis.

### Patient motivation to start using mHealth amongst patients with high emergency care utilisation

In total, 16 GUCH patients had high care utilisation. Median age was 46 years, 56% were female and 87% were symptomatic. Of all 202 GUCH patients, 25% had a mild CHD, 50% a moderate and 25% a severe CHD. Antiarrhythmic drugs were used by 69% of patients and diuretics by 44%.

Of all patients with high care utilisation, 87% were in possession of a smartphone and 18% claimed to use mHealth already. Of all patients, 44% wanted information about their disease, while 44% wanted lifestyle advice via mobile technology. A total of 56% were willing to fill in vital signs on their smartphone, 56% were willing to fill in symptoms on their smartphone, 62% wanted advice in case of aberrant vital signs, 62% wanted advice regarding symptoms of possible cardiac origin and 75% were willing to start using mHealth.

**Fig. 2:**
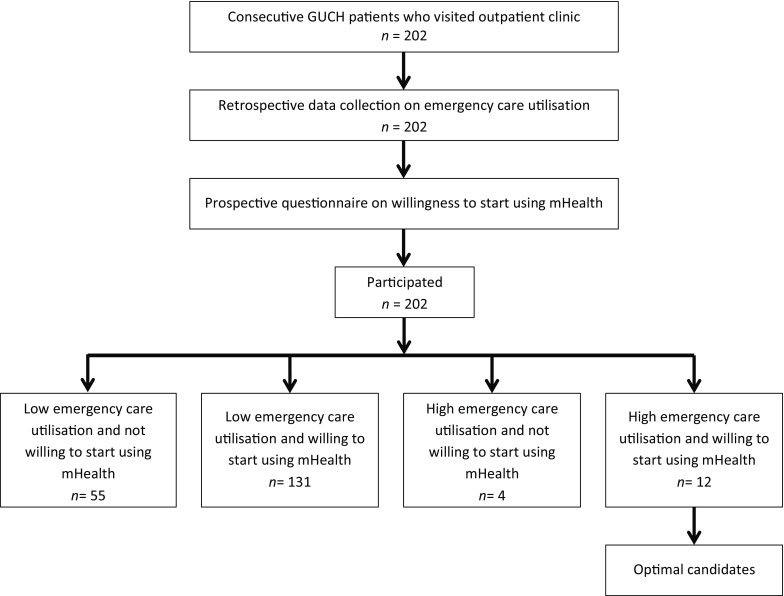
Flow chart of patient selection. *GUCH* grown-up patients with congenital heart disease

In contrast, in the low care utilisation group, 131 (70%) patients were willing to start using mHealth (Fig. 2).

## Discussion

To our knowledge this is the first study to determine the suitability of candidates for enrolment in new mHealth initiatives in GUCH patients. In our study, we found that symptomatic patients who are on diuretics or antiarrhythmic drug therapy are more likely to visit the emergency room. These patients might benefit from mHealth, as emergency visits could be prevented via mHealth. In patients with few emergency visits, mHealth is less likely to be beneficial as it is a priori less likely to prevent an emergency visit. Therefore, our study could help to avoid initiation of mHealth with the goal of decreasing emergency care utilisation in an inappropriate patient population and could prevent unnecessary data collection for patients. Furthermore, the therapeutic regimen was not changed at 44% of all emergency visits. The number of such visits might also be reducible via mHealth.

### Emergency care utilisation

In this study, 29% of all participating GUCH patients had had an emergency visit in the previous 5 years. This percentage was lower than in the study of Mackie et al. [16] and that of Verheugt et al. [17], who reported that 68% and 50% of their study population had had an emergency visit, respectively. Definitions of emergency care utilisation between Mackie et al., Verheugt et al. and our study were comparable. It is therefore hypothesised that this difference is due to the fact that for our study, only emergency visits at the Academic Medical Centre were analysed. The Academic Medical Centre is a tertiary hospital, treating patients from a large geographic region. In emergency cases, these patients are more likely to visit a local hospital close to their homes. These emergency visits are not counted in this study. Therefore, the frequency of emergency visits could be higher in our study population. In our study most patients presented with palpitations and chest pain. Arrhythmias were the most common final diagnosis. Heart failure was diagnosed in only 1% of patients, which was lower than in the studies of Cedars et al. [18] and Negishi et al. [19]. There are several explanations for this difference. First, patients might have been admitted to other hospitals. Second, in our study, diagnoses were classified according to primary diagnosis. Some patients with arrhythmias presented with heart failure symptoms but were diagnosed in the ‘arrhythmia category’. Third, two nurse practitioners specialised in heart failure had optimised treatment at the outpatient clinic, which could potentially have led to a reduction of deteriorations in heart function. Finally, in our study population, 31% had been hospitalised in the previous 5 years. This was in line with the study of Mackie et al. [16] and that of Moons et al. [20].

### Selecting GUCH patients for mobile health

Our study showed that the majority of patients were willing to use mHealth applications. Several validated technologies that allow for remote electrocardiogram (ECG) monitoring and automatic transmission are already available [21] and easy to use. For the selection of the best candidates for possible future mHealth initiatives inclusion criteria should be: GUCH patients, experiencing frequent palpitations and/or chest pain, able to operate a smartphone and having high care utilisation. Furthermore, having severe CHD, using diuretics and/or antiarrhythmic drugs, having an implant or experiencing symptoms can be taken into account in selecting GUCH patients. Gender and age should not be a discriminant factor. Issues regarding privacy will need to be addressed, since this new technology will be sensitive as regards breach of privacy. Lastly, mHealth literacy is an important predictor of success in mHealth intervention [22]. Therefore, acceptability should be taken into account when initiating mHealth initiatives in this group.

Currently, several devices that allow a user to record an ECG are already available. These devices can be used by patients themselves and do not necessitate the assistance of trained healthcare staff. As the majority of patients presented with palpitations or chest pain, mobile ECGs might contribute to improving care in these patient populations. In this study, the majority of patients with palpitations had a change in medical therapy. Innovations in the delivery of medication, for example the pill-in-the-pocket, might facilitate initial treatment at home. As such, the use of e‑Health for remote diagnosis is worth investigating.

### Limitations

This study was limited by the fact that data collection was done in a single tertiary medical centre, which could potentially affect generalisability. No data from other hospitals were incorporated in this study. Therefore, data on healthcare utilisation presented in this study might be an underestimation, as GUCH patients that participated could have been admitted to other hospitals. Lastly, 16 patients in our study had a high emergency care utilisation. This sample size is relatively small and the percentages derived from this sample should therefore be interpreted with caution.

### Planned healthcare utilisation

This study was primarily concerned with the role of mHealth to decrease emergency care utilisation. It might, however, be possible that frequent collection of vital signs and remote doctor-patient contact will decrease the need for planned in-office visits as well. Moreover, mHealth could also contribute to the improvement of patient satisfaction and patient health engagement [23]. This should be measured in future mHealth initiatives as well.

## Conclusion

GUCH patients who are symptomatic, those on antiarrhythmic drug therapy and those on diuretics are optimal candidates for enrolment in new mHealth initiatives because of both a high care utilisation and high motivation. Our study contributes to appropriate patient selection for mHealth initiatives that aim to prevent emergency care utilisation, thereby contributing to an efficient use of mHealth.
